# An Enhanced Dissolving Cyclosporin-A Inhalable Powder Efficiently Reduces SARS-CoV-2 Infection In Vitro

**DOI:** 10.3390/pharmaceutics15031023

**Published:** 2023-03-22

**Authors:** Davide D’Angelo, Eride Quarta, Stefania Glieca, Giada Varacca, Lisa Flammini, Simona Bertoni, Martina Brandolini, Vittorio Sambri, Laura Grumiro, Giulia Gatti, Giorgio Dirani, Francesca Taddei, Annalisa Bianchera, Fabio Sonvico, Ruggero Bettini, Francesca Buttini

**Affiliations:** 1 Food and Drug Department, University of Parma, Parco Area delle Scienze 27a, 43124 Parma, Italy; 2 Department of Experimental, Diagnostic and Speciality Medicine, University of Bologna, 40138 Bologna, Italy; 3 Microbiology Unit, The Great Romagna Area Hub Laboratory, Piazza della Liberazione 60, Pievesestina, 47522 Cesena, Italy

**Keywords:** cyclosporine-A, spray-drying, dry powder inhaler, SARS-CoV-2, cytokine storm, transplant rejection

## Abstract

This work illustrates the development of a dry inhalation powder of cyclosporine-A for the prevention of rejection after lung transplantation and for the treatment of COVID-19. The influence of excipients on the spray-dried powder’s critical quality attributes was explored. The best-performing powder in terms of dissolution time and respirability was obtained starting from a concentration of ethanol of 45% (*v*/*v*) in the feedstock solution and 20% (*w*/*w)* of mannitol. This powder showed a faster dissolution profile (Weibull dissolution time of 59.5 min) than the poorly soluble raw material (169.0 min). The powder exhibited a fine particle fraction of 66.5% and an MMAD of 2.97 µm. The inhalable powder, when tested on A549 and THP-1, did not show cytotoxic effects up to a concentration of 10 µg/mL. Furthermore, the CsA inhalation powder showed efficiency in reducing IL-6 when tested on A549/THP-1 co-culture. A reduction in the replication of SARS-CoV-2 on Vero E6 cells was observed when the CsA powder was tested adopting the post-infection or simultaneous treatment. This formulation could represent a therapeutic strategy for the prevention of lung rejection, but is also a viable approach for the inhibition of SARS-CoV-2 replication and the COVID-19 pulmonary inflammatory process.

## 1. Introduction

Cyclosporine-A (CsA) is a cyclic peptide with an immunosuppressive action, administered for the treatment of various pathologies that share uncontrolled activation of the immune system, e.g., atopic dermatitis and psoriasis. Since entering the market in 1983, CsA, a calcineurin inhibitor peptide, has been widely used in the treatment of various autoimmune conditions characterised by the strong activation of the immune system [1]. The success of this molecule is related to its selective and reversible inhibition of the production of pro-inflammatory cytokines by T-lymphocytes [2]. CsA is intravenously and orally (as soft capsules) administered and is currently used for the prevention of allograft rejection in various organ transplantations. Indeed, the continuous activation of T-cells in the transplanted lung is the key factor bringing on bronchiolitis obliterans syndrome (BOS) characterised by extensive fibroproliferation and loss of lung functionality [3]. BOS is considered a marker of chronic rejection and causes 30% of deaths after lung transplantation [4,5].

Despite the efficacy of CsA, severe adverse side effects including nephrotoxicity, hepatotoxicity, hypertension, and neurotoxicity, usually arise during chronic treatment with CsA [6,7]. Moreover, the delivery of a sufficient and reproducible amount of CsA can hardly be achieved by oral administration because of its poor aqueous solubility, its pre-systemic metabolism at the gut level [8,9], and its erratic absorption related to interindividual variability, food intake, and by comorbidities such as diabetes [10,11]. Overall, the oral bioavailability of CsA is around 30%, which entails a dosage range between 5 and 15 mg/kg/day, and the need to carefully monitor the patient’s drug plasma concentration over time [12].

For this reason, pulmonary administration would be a promising strategy for the treatment of lung transplant patients, given the possibility of (i) avoiding pre-systemic metabolism and obtaining high drug local concentrations, (ii) having a rapid onset of action, and (iii) administering lower doses than the oral route with limited systemic exposure to the drug. In this regard, the administration of a 100 µg intratracheal dose of CsA to rats, in addition to being effective in reducing lung inflammation, led to a distribution of CsA in the side effect-related organs that were one hundred times lower than that of an oral dose of 10 mg/kg [13]. The pulmonary administration of a dose of just 5 mg of CsA by nebulisation of a propylene glycol solution was able to produce an improvement in lung-transplanted patients’ conditions, expressed as forced expiratory volume in one second (FEV1) [14]. This study demonstrated a strong relationship between the administration of CsA directly to the lungs and an increased anti-rejection effect. Further clinical trials have confirmed the benefits of direct pulmonary administration of CsA by nebulisation in patients who underwent single or double lung transplantation [15] or in BOS patients [16].

Besides the effect on the prevention of allograft rejection, CsA has also been widely studied as a potential anti-viral drug [17,18,19]. In 2011, de Wilde and colleagues first demonstrated the in vitro inhibitory activity of CsA at micromolar concentrations on the replication of different coronavirus genera [20]. The effective inhibition of replication towards SARS-CoV-2 has also recently been demonstrated by Fenizia et al. on the human lung epithelium Calu3 cell line [19]. In addition, CsA anti-inflammatory and immunomodulatory activities would be beneficial in containing the cytokine storm experienced by many COVID-19 patients, leading to airway damage and respiratory loss of function [19,21].

COVID-19 is currently treated using antiviral drugs such as molnupiravir [22], nirmatrelvir [23], ritonavir [24], and remdesivir [25], anti-inflammatory drugs (dexamethasone [26]), immunomodulatory agents such as baricitinib and tocilizumab, an anti-IL-6 antibody [27], and monoclonal antibodies against the receptor binding domain such as sotrovimab [28].

The aim of this work was the development of a highly respirable formulation of CsA obtained by spray drying with excipients already approved for inhalation. A critical parameter for the evaluation of the quality of the produced powders was the Weibull dissolution time obtained from the in vitro release rate profile. The most promising powder was then further analysed in terms of tolerability, reduction of inflammation, and antiviral activity in terms of SARS-CoV-2 reduction of infection in Vero E6 cells.

## 2. Materials and Methods

CsA (Metapharmaceutical, Barcelona, Spain) was purchased from ACEF (Fiorenzuola d’Arda, Italy). HPMC extra-dry capsules for use in dry powder inhalers, Quali-V^®^-I size #3, were provided by Qualicaps (Madrid, Spain), while the high-resistance dry powder inhaler RS01 was gifted by Plastiape (Lecco, Italy). Mannitol was purchased from Roquette (Lestrem, France) and glycine was purchased from Sigma Aldrich (Merck, Milano, Italy). All other chemicals used were obtained from commercial suppliers and were at least of analytical grade. The human lung adenocarcinoma cell line A549 (CRM-CCL-185), monocytic cell line THP-1 (TIB-202), and Vero E6 (CRL-1586) were purchased from American Type Culture Collection (ATCC, Manassas, VA, USA).

### 2.1. Preparation of CsA Spray-Dried Powders

The spray-dried (SD) CsA powders were obtained starting from a solution of water and ethanol 96% with a variable ratio according to the design of experiment (DOE) containing 1% (*w*/*v)* solids. The effect of different amounts of excipients on the yield of production, respirability, residual solvent, and dissolution rate was assessed. The experiments were designed by means of the Design-Expert 12 software (Stat-Ease, Inc., Minneapolis, MN, USA). A half-fractional factorial design with three factors at two levels and three additional centre points for curvature check was applied, requiring a total of 11 experiments, as detailed in Table 1. The mannitol (10–20% *w*/*w*) and glycine (0–5% *w*/*w*) content in the dry formulation and ethanol (45–60% *v*/*v*) concentration in the feed solution were the three factors investigated, fixed at two levels equally distant from the central point.

CsA raw material (CsA_rm) was solubilised in ethanol where the solubility of CsA is more than 100 mg/g [29], while mannitol and glycine were solubilised in water at room temperature. The aqueous solution was added to the CsA solution under magnetic stirring (160 rpm). The CsA remained in solution in all the ranges of water added (from 40 to 55 % *v*/*v*) in the hydroalcoholic solution.

To produce the powders, 50 mL solution was spray dried (Mini Spray Dryer B-290, Büchi, Flawill, Switzerland) using the following parameters: inlet temperature 140 °C, drying air flow rate 742 L/h, aspiration 35 m^3^/h, solution feed rate of 3.5 mL/min, and a nozzle diameter 0.7 mm. Under these conditions, an outlet temperature of 80–87 °C was measured.

An analysis of variance (ANOVA) was performed to investigate the effect of factors on the critical quality attributes (CQAs). In detail, the CQAs selected were the production yield, the percentage of residual solvent, and the Weibull dissolution time obtained from the dissolution profile. The probability value of the model was considered significant when lower than 0.05.

### 2.2. CsA Quantification by High-Performance Liquid Chromatography (HPLC)

The quantification of CsA in the spray-dried powders was achieved by dissolving 20 mg of powder in 25 mL of water:acetonitrile 40:60. Six samples were prepared and analysed by HPLC. The drug content analysis was conducted after the powder preparation and during the stability study.

CsA was quantified using an HPLC (LC-10, Shimadzu, Kyoto, Japan) equipped with a UV–Vis detector, set at a wavelength of 230 nm and using the column Nova-Pak C18 (3.9 × 150 mm, 4 µm; Waters, Italy). The mobile phase was constituted by a mixture of 65% acetonitrile and 35% ultrapure water, acidified at 0.1% with trifluoroacetic acid. The column temperature was set at 65 °C and the flow rate was fixed at 1.6 mL/min. The injection volume was 10 µL, the run time of was 10 min and the retention time for the CsA was about 5 min. The method linearity was over the range 0.1–2 mg/mL. 

### 2.3. Aerodynamic Performance Characterisation

The screening of the aerodynamic performance of all CsA batches produced was achieved using Fast Screening Impactor (FSI; Copley Scientific, Nottingham, UK), with a 65 L/min insert to provide a 5 μm cut-off size. The FSI was connected to an SCP5 vacuum pump (Copley Scientific, Nottingham, UK) through a critical flow controller (TPK Copley Scientific, Nottingham, UK). A flow rate of 65 L/min, measured with a DFM 2000 Flow Meter (Copley Scientific, UK), was required to activate the RS01 (Plastiape, Lecco, Italy) device at a 4 kPa pressure drop. The TPK actuation time was adjusted so that a volume of 4 L of air was drawn through the inhaler. The content of one capsule, filled with 20 mg of powder, was discharged and each experiment was repeated three times. The amount of CsA present in the formulation was in the range between 15 to 18 mg according to the formulation drug content. CsA was quantified by HPLC using to the method reported in Section 2.2. The emitted fraction (EF) was calculated as the percentage ratio between the total CsA mass recovered in FSI and the CsA loaded in the capsule. The respirable fraction (RF) was calculated as the percentage ratio between the mass of particles with an aerodynamic diameter less than 5 µm and the emitted dose.

The same analysis setup was maintained to further investigate the aerodynamic performance using the Next Generation Impactor (NGI; Copley Scientific, Nottingham, UK). To obtain a more accurate analysis and avoid the eventual particles bouncing, the cups of the impactor were coated using a solution of 2% (*w*/*v*) Tween 20 in ethanol. As above, the content of one capsule of 20 mg was aerosolised and the CsA in the NGI was collected and quantified by HPLC.

The metered dose (MD) is the total mass of the drug, quantified by HPLC, recovered in the inhaler and the impactor (induction port, stages 1 to 7, and Micro Orifice Collector (MOC)). The emitted dose (ED) is the amount of drug leaving the device and entering the impactor (induction port, stages 1 to 7, and MOC). The mass median aerodynamic diameter (MMAD) was determined by plotting the cumulative percentage of mass less than the stated aerodynamic diameter for each NGI stage from 1 to 7, on a probability scale versus the aerodynamic diameter of the stage on a logarithmic scale. The fine particle dose (FPD) is defined as the mass of drug with an aerodynamic diameter less than 5 μm (calculated from the log-probability plot equation) and the extra fine particle dose (EFPD) is the mass of the drug with an aerodynamic diameter less than 2 μm. The fine particle fraction (FPF) and the extra fine particle fraction (EFPF) were calculated as the percentage ratio between the FPD or EFPD, respectively, and the ED.

### 2.4. Thermogravimetric Analysis (TGA)

The analysis was carried out using the TGA/DSC 1 STARe System (Mettler Toledo, Columbus, OH, USA) to determine the loss on drying (LOD), i.e., the percentage of residual humidity and solvents present in the powder at the end of the manufacturing process. For this purpose, approximately 4 mg of powder was placed in a pan of aluminium oxide, and the analysis was carried out in a nitrogen flow at 80 mL/min. The temperature was increased from 25 °C to 150 °C with a rate of 10 °C/min. The LOD was measured in the range 25–125 °C.

### 2.5. Dissolution Profile of Respirable Particle Fraction

In vitro dissolution tests to compare the dissolution performance of CsA powders were conducted using RespiCell^TM^ [30], an innovative vertical diffusion cell apparatus.

The apparatus comprises a 170 cm^3^ receiving cell filled with the dissolution media, and the sampling was performed through the side arm. The apparatus constitutes two portions: the upper part acts as a donor chamber and the lower part is a receptor chamber maintained under magnetic stirring at 180 rpm.

The receptor was filled with 170 mL of medium consisting of phosphate-buffered saline (PBS) containing 0.2% of sodium dodecyl sulphate and the cell was connected to a heating thermostat (Lauda eco silver E4, DE) set at 37 ± 0.5 °C. The dissolution was carried out on the RF of the powder, following separation by FSI. In the case of spray-dried CsA, four capsules of 20 mg were aerosolised for each experiment and the analysis was performed in triplicate. In the case of the raw material, the content of ten capsules was aerosolised due to the low respirability of the material. The filter (Type A/E glass filter 7.6 cm diameter, Pall Corp.) containing the mass of powder < 5 µm was then placed on the diffusion area of the RespiCell and 2 mL of PBS containing 0.2% of SDS was added before starting the dissolution to create a thin liquid layer on the powder bed. At fixed intervals, 1 mL of the receiving solution was removed and replaced with 1 mL of fresh buffer to maintain a constant volume inside the receptor chamber.

Finally, at the end of the experiment, the residual undissolved powder was recovered by washing out the filter with 10 mL of ethanol:water (50:50 *v*/*v*). The samples were quantified by HPLC according to the method described. The drug dissolved was expressed as a percentage of CsA dissolved relative to the total CsA recovered at the end of the test both on the filter and receptor compartment. 

The dissolution profiles were analysed by means of the Weibull equation [31] in order to determine the time parameter, recognised as the time at which the 63.2 per cent of the drug was dissolved.

### 2.6. Morphological Analysis by SEM

Particle morphology was determined by scanning electron microscopy (SEM, Zeiss AURIGA, Zeiss, Oberkochen, Germany) and was operated under high-vacuum conditions with an accelerating 1.0 kV voltage at a magnification of 5k times. The powders were deposited on adhesive black carbon tabs pre-mounted on aluminium stubs and imaged without undergoing any metallisation process.

### 2.7. Viability Study on A549 and THP-1

A549 cells (seeding 10^4^ cells/well), following overnight culture, and THP-1 cells (seeding 5 × 10^4^ cells/well), immediately after seeding in a 96-well plate at 37 °C, were exposed to the following treatments: vehicle (DMSO 0.5% in PBS), CsA_rm (1, 10 µg/mL), spray-dried powder CsA_M20 (containing 20% *w*/*w* of mannitol) (1, 10 µg/mL of CsA), and mannitol 2 µg/mL. Cell viability was quantified using the MTS assay. Briefly, 20 μL of 3-(tributylammonium)-propyl methanethiosulfonate bromide solution (MTS, 1 mg/mL) was added to each well and, following 4 h incubation at 37 °C, the supernatants were collected. The absorbance of each well was measured at 490 nm on a microplate reader (Sunrise™ powered by Magellan™ data analysis software, TECAN, Mannedorf, Switzerland). The impact of the various treatments on cell viability was expressed as the percentage of viability with respect to vehicle-treated cells.

### 2.8. Co-Culture Assays and Cytokine Determination

For the co-cultures, A549 cells (10^5^ cells/well) were seeded at the bottom and THP-1 cells (10^5^ cells/well) were plated on the insert (0.4 μm pore polyester filter) of Transwell culture plates (#3470, Corning Inc., Corning, NY, USA), with the two cell cultures being physically separated to avoid direct contact, according to the method described by Li et al. [32]. After 24 h co-culture, the cells were exposed to the following treatments: vehicle (DMSO 0.5% in PBS), CsA_rm 10 µg/mL, CsA_M20 at 10 µg/mL in respect to CsA, mannitol 2 µg/mL in DMSO 0.5% in PBS. After 1 h, LPS 1 μg/mL (*Escherichia coli* O55:B5; cat# L6529; Sigma Aldrich, Merck, Milano, Italy) was added to the culture and maintained for 24 h. Cells incubated with the vehicle and not exposed to LPS were used as the control. The concentration of IL-6 in the conditioned media was subsequently determined using an ELISA kit (Boster Biological Technology, Milano, Italy; cat. no. IL-6, EK0410), according to the manufacturer’s protocol and expressed as pg/mL.

### 2.9. Cell Treatment and Viral Replication Inhibition Assay

The inhibitory effect of CsA_M20, CsA_rm, and mannitol on viral replication on Vero E6 cell cultures was tested against Omicron subvariant BA.1 (lineage B.1.1.529.BA.1). 

The viral strain was isolated from a residual clinical specimen conferred to the Unit of Microbiology, Greater Romagna Area Hub Laboratory (Cesena, Italy). The sample underwent an anonymisation procedure in order to adhere to the regulations issued by the local Ethical Board (AVR-PPC P09, rev.2; based on Burnett et al., 2007 [33]). Detailed descriptions of Vero E6 cell culture and propagation, as well as titration and isolation of the virus from biological samples, are reported in the Supplementary Materials.

The day before treatment and infection, Vero E6 cells were seeded at a density of 2 × 10^6^ cells/well in 96-well plates and allowed to attach for 16 to 24 h at 37 °C, 5% CO_2_. On the day of infection, each tested compound stock suspension in PBS was freshly diluted in cell culture medium containing 2% FBS. CsA_rm was tested at concentrations of 8, 16, 32, and 64 µM, corresponding to 9.6, 19.2, 38.4, and 76.9 µg/mL; CsA_M20 was diluted to obtain the same CsA concentrations considering the exact CsA content in the powder (determined by HPLC) of about 80% *(w*/*w)*. The selected CsA concentrations, in the case of powder CsA_M20, involved the presence of dissolved mannitol at concentrations of 2.4, 4.8, 9.6, and 19.2 µg/mL since mannitol represents 20% (*w*/*w)* of the formulation. These values were then adopted when mannitol was applied to the cells and tested as vehicle alone.

To better determine at which level the viral replication cycle was inhibited, the cells were subjected to different treatment regimens: treatment 1 h before infection (pre-treatment), treatment 2 h after infection (post-infection), and treatment during infection (simultaneous). Each treatment lasted one hour then was removed. Antiviral efficacy was tested against the viral concentration of 0.0005 moi. Infected cultures were incubated for one hour at 37 °C to allow viral adsorption then the supernatant was removed, and cells were washed with PBS. Treated and infected cultures, were incubated with cell medium at 37 °C, 5% CO_2_ for 72 h. For each treatment protocol, the cell culture was infected directly with the virus suspension to assess viral replication in the absence of any potential inhibition. 

### 2.10. SARS-CoV-2 Nucleic Acid Quantification 

Viral replication in treated and untreated cell cultures was evaluated by qRT-PCR by comparing the cycle threshold (Ct) values of each treated sample (Ct treated) and its corresponding untreated control (Ct control) obtained after 72 h of incubation. For this purpose, the Allplex SARS-CoV-2 Extraction-Free system (Seegene Inc., Seoul, South Korea) was used. It consists of a real-time qRT-PCR multiplex assay based on the use of TaqMan probes. The sample preparation, reaction setup, and analysis were performed according to the manufacturer’s instructions and the details are described in the Supplementary Materials. Positive and negative controls were included in each run. Fluorescent signals were acquired after every amplification cycle. By comparing the Ct values referring to the N-gene of each treated sample and its corresponding untreated control obtained at the end of the test, the percentage of infectivity reduction was calculated, as follows:

$$\%\;\mathrm{viral}\;\mathrm{infectivity}\;\mathrm{reduction}=\frac{\mathrm{Ct}\;\mathrm{treated}-\mathrm{Ct}\;\mathrm{control}}{{\mathrm{Ct}}_{0}-\mathrm{Ct}\;\mathrm{control}}*100$$

where Ct_0_ represents the cycle threshold at the time of treatment application.

Cells treated with the same treatment protocols, but not infected, were used to assess the effects on cell viability. To quantify cell viability, after the incubation period, the cell monolayers were fixed and stained using 4% formaldehyde solution in crystal violet; absorbance was read at 595 nm. For each tested compound concentration, the percentage of viable cells for each tested concentration was calculated, setting the mean absorbance value of the cell control wells (neither treated nor infected cells) as 100% viability. None of the CsA_M20, CsA, or mannitol concentrations significantly compromised the cell viability.

### 2.11. Stability Studies

Stability studies were conducted on CsA_M20 spray-dried powder by storing the capsules containing 20 mg of powder at 25 °C and 60% of relative humidity (RH) and 40 °C and 75% of RH. The CsA content and in vitro aerodynamic performance by NGI were studied after 1 and 3 months of storage.

### 2.12. Statistical Analysis

Statistical analysis was conducted using the analysis of variance (ANOVA test) with a post hoc test using Prism 9 (GraphPad Software, v.9.4.0). Data were considered to be statistically significant when the *p*-value was < 0.05 (* = *p* < 0.05; ** = *p* < 0.01).

## 3. Results and Discussion

### 3.1. CsA Dry Powder Development by DOE

CsA is a lipophilic molecule with a logP of 3 and poor water solubility (3.69 mg/L at 37 °C), falling into class II of the Biopharmaceutical Classification System (BCS) among molecules with low water solubility and high permeability [34]. These physico-chemical properties limit the bioavailability of CsA, and many studies have been performed to improve the dissolution profile of CsA including the use of nanoparticles incorporated into microparticles by spray drying or spray freeze drying [13,35,36].

Moreover, the direct deposition of CsA to the lung could be an effective strategy in preventing lung rejection due to the high local drug availability also enhanced by the avoidance of intestinal pre-systemic metabolism.

The low water solubility of CsA represents an issue for the development of an inhalation product both from the point of view of the formulation and the release of the drug on site. In the case of a nebulisation product, a CsA solution using propylene glycol as a solvent [14] or a liposomal formulation has been proposed to increase the pulmonary exposure of the drug. Despite the good performance in clinical trials, the CsA solution for nebulisation did not reach the market, perhaps because of the possible irritant effect of the solvent used [37,38]. Other clinical trials conducted using inhaled liposomal CsA demonstrated the capability of the drug to increase BOS-free survival [39,40].

Compared to a CsA liquid nebulisation, the use of a CsA inhalation powder offers numerous advantages: the powder can be administered by a quick inhalation act and, as a solid-state formulation, the stability of the product is increased. On the other hand, the development of a powder containing CsA requires particular attention to be paid to the choice of excipients and the production technique capable of improving the release of the drug from the solid particles. In this context, some strategies have been proposed to enhance pulmonary release and absorption, such as the construction of CsA particles with pulmonary surfactants or with hydroxypropyl-beta-cyclodextrin and hydrosoluble chitosan [41,42,43].

In this work, the spray-drying process and water-soluble excipients were chosen to develop physically stable CsA respirable particles with improved dissolution. Mannitol was selected as it is currently approved for pulmonary administration [44] and is widely used in particle engineering. The addition of glycine was investigated to promote powder deaggregation and aerosolisation.

**Figure 1:**
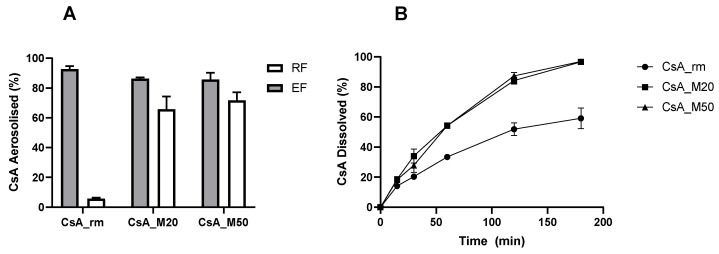
Aerosolisation performance (RF = respirable fraction, EF = emitted fraction) (**A**) and dissolution profiles (**B**) of CsA raw material (CsA_rm), CsA_M20, and CsA_M50. Data presented as n = 3, mean value ± SD.

A preliminary study was carried out to identify the most suitable amount of mannitol to add to the formulation and subsequently to keep it as a starting point for a more in-depth investigation by DOE. Figure 1A illustrates the EF and RF of powders containing CsA and mannitol in the two ratios of 80:20 (CsA_M20) and 50:50 (CsA_M50), spray-dried starting from a solution containing 45% (*v*/*v*) ethanol in water. Similar EF and RF values were shown by the two CsA–mannitol powders: the EF was around 85% and RF was about 68–70%. On the contrary, CsA_rm, which had a volume median diameter of 7.67 µm, had a large deposition in the induction port of the impactor, which led to a very low RF of 6%.

Both of the CsA spray-dried powders exhibited a faster dissolution rate than the CsA_rm: approximately 87% of the spray-dried powder was dissolved after 3 h of the experiment, while only 50% of the raw material was dissolved (Figure 1B). However, the addition of mannitol in different quantities did not lead to a difference in the release profiles of CsA_M20 and CsA_M50. This preliminary test shows that, when mannitol exceeded 20% (*w*/*w*) in the powder composition, it no longer had any positive effect on the formulation for either of the qualitative parameters studied. Hence, with the purpose of limiting the amount of powder to inhale, it was decided that the amount of mannitol in the formulation would remain fixed at 20%. 

A screening DoE was set up to investigate the influence of excipients on the quality of the powders. The effect of the ethanol content in the feedstock solution and the addition of glycine along with mannitol on the CQAs of the powders were investigated and are illustrated in Table 2. The yield of the process and the loss on drying (LOD) describe the quality of the spray-drying process, whereas the powder’s aerodynamic behaviour (i.e., RF) and the dissolution time are related to the quality of the formulation. The residual solvent in the dried powder could affect not only its chemical stability, but also its respirability over time, as it could modify the powder’s properties.

An ANOVA of the responses for the selected factorial model was performed. The generated model was not significant for the yield of production and for the respirable fraction. In fact, the yield value was similar for all powders, regardless of the composition of the stock solution. In general, the results indicate that the process was efficient in terms of the amount of powder produced and was robust. The yield of the manufacturing process was in the range of 55–65% for all powders. In all cases, the microparticles did not give rise to visible aggregates and the powders were not electrostatic. Not only the process was considered robust with acceptable values, but also, regarding the respirable fraction, the composition of the feed solution did not have a significant impact within the investigated ranges.

**Figure 2:**
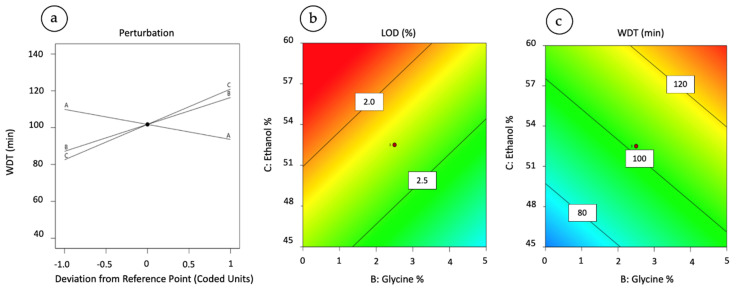
Perturbation graph of Weibull dissolution time (WDT) versus the critical factors plotted as deviation from the reference point (A = mannitol; B = glycine; C = ethanol) (**a**). Contour plot of LOD (**b**) and WDT (**c**) as a function of ethanol and glycine proportion in feed solution at the mannitol concentration of 15% (*w*/*w*).

Conversely, the ANOVA revealed that the model was significant for the LOD and WDT with probability values of 0.033 and 0.006, respectively (Table 3). Furthermore, the robustness of the relationship between the model and the variables analysed was high, as indicated by the R^2^ values. Figure 2 illustrates the perturbation graph of WDT versus the three critical factors and contour plot of LOD and WDT as a function of ethanol and glycine proportion.

Ethanol is the main factor influencing the different degrees of residual solvents in the particles. As the percentage of ethanol increases, the LOD value approaches zero per cent. On the contrary, glycine had a negative effect on the powder LOD: the presence of this excipient increased the amount of residual solvent in the powder; hence, it was not beneficial for the formulation quality aspects. According to this model, the percentage of mannitol also positively influences the LOD; however, this would seem to be a parameter deriving from the combined effect of ethanol and glycine. A low LOD value is important because it usually correlates with improved peptide stability in a solid-state formulation and decreases the possibility of mannitol recrystallisation. The graph in Figure 2b illustrates the trend of the LOD as the ethanol and glycine vary.

The main contribution to the variation in the dissolution time is due to glycine and ethanol, while the effect of mannitol was not significant. Therefore, as the percentage of ethanol and glycine increases, the time to dissolve the 63.2% of the API rises (see Figure 2c). Mannitol did not have a statistically significant effect, although it was indicated as a factor reducing WDT, i.e., leading to a faster dissolution rate (see Figure 2a). 

In general, the spray-drying process was always able to produce particles with an enhanced dissolution rate compared to the non-formulated CsA (WDT of 169.0 min). Among all of the formulations, the powder CsA_M20, which was prepared starting from a feed solution containing 45% ethanol and without glycine, had the lowest WDT of approximately 59 min. The drug release profile of CsA_M20 was similar to that obtained by Yamasaki et al. (WDT of approx. 62 min) when CsA was precipitated in nanoparticles and spray-dried into nano-matrix structures with lactose mannitol and lecithin [35]. However, although the dissolution profile of the engineered powders was improved compared to the raw material, it is still a rather slow dissolution rate, which places undissolved particles at risk of removal by mucociliary clearance or phagocytosis. Therefore, in vivo studies will be useful to fully prove the beneficial effect of such formulations.

The observed behaviour indicated that when the particle composition consisted only of mannitol and CsA, this was more favourable for dissolution and in terms of residual solvent content. The reason why the composite CsA particles have a higher dissolution rate than the raw material is because during particle formation, the mannitol precipitates together with the CsA, forming a solid structure where the two materials are intimately dispersed. In contact with an aqueous medium, the mannitol dissolves immediately, leaving the CsA, with a high surface area, free for dissolution. Interestingly, the presence of glycine lowered the release of CsA, although it is a hydrophilic excipient, but less hygroscopic than mannitol. 

Given the significance of the data, it will be worthwhile to further investigate the effect of the interactions between the factors and the CQAs using a full factorial DOE.

**Figure 3:**
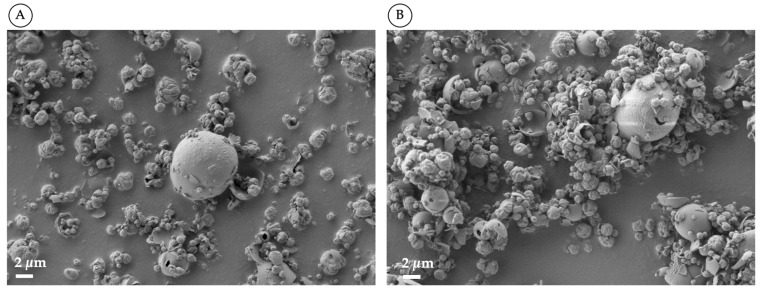
SEM images of CsA spray-dried powders produced with 45% (*v*/*v)* (**A**) and 60% (*v*/*v)* (**B**) of ethanol in the feedstock solution.

The ethanol content of the feed solution also influenced the morphology of the microparticles obtained. When the ethanol was 45% (Figure 3A), the particles appeared to be less inflated and more corrugated than the particles produced from a solution containing 60% ethanol (Figure 3B), where a greater number of large, fractured particles were observed. This behaviour is in agreement with what was reported for the production of amikacin spray-drying powders [45]: the particles are much larger or exploded when the evaporation rate is rapid, and therefore the precipitation of the solute occurs early. The evaporation rate increases as the percentage (*v*/*v*) of ethanol in the feed solution rise. 

With regard to the solid state of the produced CsA powders, all were amorphous, as evidenced by the typical halo of the X-ray pattern (see Supplementary Materials). The structure of the CsA raw material was also amorphous before spray drying and no crystallinity peaks were observed in the powders after production.

From this first part of the work, CsA_M20 was selected as the best-performing powder and was then further characterised and tested for its tolerability, anti-inflammatory and antiviral activity. 

### 3.2. Full Characterisation of the CsA_M20 Spray-Dried Powder

The CsA drug loading in the CsA_M20 powder after its production was 76.3 ± 1.4%. This value agreed with the theoretical one (80%) considering that the powder had a solvent content, determined by TGA, of about 2%.

The aerodynamic particle size distribution of the powder CsA_M20, assessed by NGI, showed that the formulation had a very high respirability. The emitted amount of powder from the RS01 device was 16 mg (corresponding to 90% of the metered dose) containing 13.2 mg of CsA. The FPD was 8.8 mg of CsA, which corresponds to an FPF of 66.5 % (Table 4). The favourable aerodynamic behaviour can be attributed both to the poor cohesiveness of the particles and their good flowability and to the efficient deaggregation mechanism of the RS01 device.

**Figure 4:**
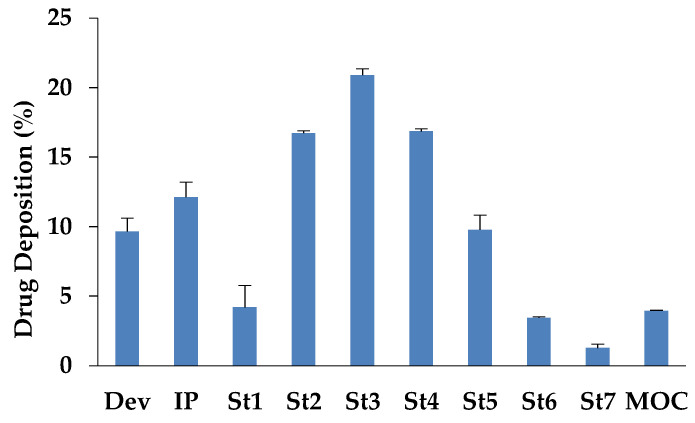
Distribution of CsA_M20 powder on Next Generation Impactor. The loaded amount of powder in the capsule was 20 mg containing 16 mg of CsA, (n = 3, mean value ± SD). Dev = device; IP = induction port; St = stage; MOC = micro-orifice collector.

From Figure 4, illustrating the deposition of the CsA in the NGI, it is possible to observe that most of the particles were collected in stages 2, 3, and 4 and about 4% was collected in the MOC capturing particles with a size lower than 0.5 µm. This led to obtaining an MMAD value of 2.97 µm. 

A clinical trial evaluating the CsA anti-inflammatory efficacy in BOS by the nebulisation of 300 mg demonstrated that a deposition of CsA greater than 5 mg in the lung correlates with an improvement in lung functionality, and 12 mg was indicated as an anti-rejection protective dose [14]. In light of these results, it can be considered that the FPD of 8.8 mg, generated by the aerosolisation of 20 mg of CsA_M20, is in the correct therapeutic range for the prevention of BOS.

Regarding the management of the COVID-19 infection, there are no efficacy or pharmacokinetic data upon the delivery of CsA by inhalation. However, COVID-19 patients who received 300 mg of CsA orally showed positive results on survival [46]. At this dose, the amount of CsA available to the lung will have been very low, but still sufficient to dampen the inflammatory reaction of the respiratory tract. Inhalation administration would make it possible to obtain equal or higher efficacy in the face of a reduction in the administered dosage and reduced systemic exposure. 

Stability analyses on the CsA_M20 powder stored in HPMC capsules, conducted at 1 and 3 months in standard and accelerated conditions, provided drug content values in a range between 78 and 82% without being significantly different from the time zero (*p* < 0.05). In the aerodynamic assessment, the CsA ED was around 13 mg and the FPD was in the range of 8–9 mg, independently of the storage conditions and the check time of the analysis (Table 4). These data, albeit preliminary, show that the use of mannitol as a bulking excipient was able to protect the physicochemical stability of the formulation, preserving its initial characteristics. The use of Quali_V^®^_I capsules in this work, specifically produced for DPI, with optimised puncturing properties and internal lubricant features, certainly contributed to this positive achievement [47]. Finally, the CsA_M20 showed a differential scanning calorimetry profile at three months equal to that at time zero, evidencing that the powder did not undergo solid-state transformations during the observation time (see Supplementary Materials).

### 3.3. CsA_M20 Cytotoxicity and Anti-Inflammatory Efficiency

**Figure 5:**
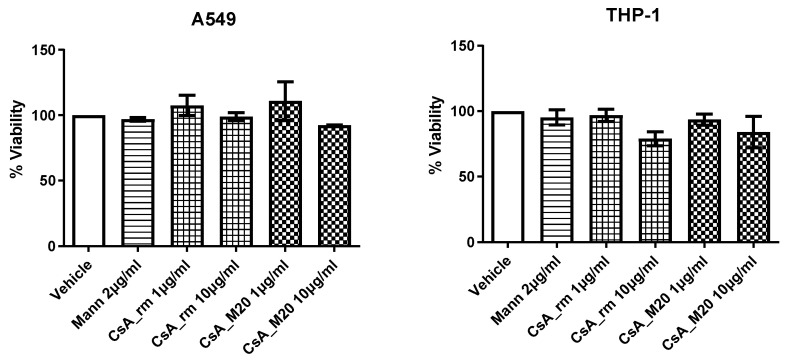
Viability of A549 and THP-1 cell cultures exposed to the vehicle, mannitol (Mann) 2 μg/mL, CsA_rm at 1 and 10 µg/mL, CsA_M20 spray-dried powder at of 1, and 10 µg/mL of CsA. Data are expressed as a percentage with respect to the vehicle.

The viability of human lung adenocarcinoma cell line A549 and monocytic cell line THP-1 was not affected by the various CsA tested treatments, which were well tolerated by cells, as reported in Figure 5. Indeed, under these conditions, neither CsA_rm nor the spray-dried powder of CsA containing mannitol displayed any cytotoxic effect on the two cell cultures compared to the vehicle (0.5% DMSO in PBS).

IL-6 is a pro-inflammatory cytokine involved in numerous cellular processes such as proliferation and survival. Furthermore, the high serum levels of IL-6 in patients who have undergone a lung transplant were a marker for the development of chronic lung allograft dysfunction [48,49]. In parallel, it was observed that COVID-19 infection is accompanied by an aggressive inflammatory response with the release of a large amount of pro-inflammatory serum cytokines in an event known as a “cytokine storm” [50]. In particular, IL-6 was reported to be a potential predictor for the development of severe COVID-19, since elevated levels of this cytokine were associated with critical patient conditions such as acute respiratory distress syndrome and the need for mechanical ventilation [50]. As IL-6 is the most frequently reported cytokine to be increased in COVID-19 patients and as IL-6 elevated levels have been associated with higher mortalities, this cytokine was selected in this work to test the CsA anti-inflammatory effect.

**Figure 6:**
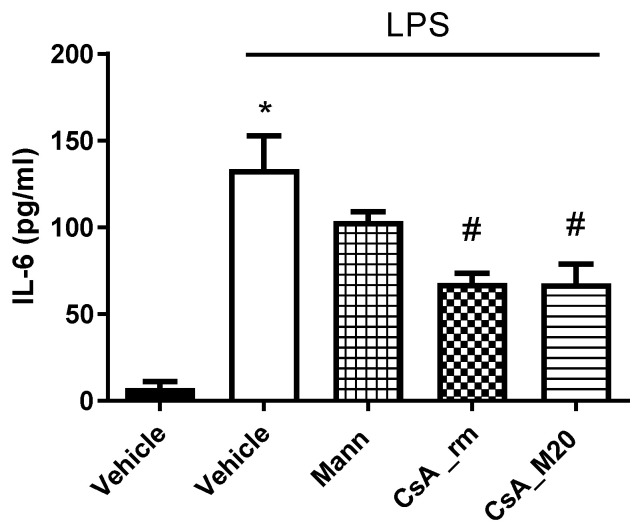
In vitro inhibition of IL-6 production by THP-1/A549 co-culture exposed to LPS in the presence of the vehicle, CsA_rm at 10 µg/mL, CsA_M20 at 10 µg/mL of CsA, or mannitol (Mann) at 2 μg/mL; * *p* < 0.05 vs. vehicle; # *p* < 0.05 vs. vehicle + LPS, ANOVA test followed by Bonferroni’s post-test.

The levels of IL-6 were determined by ELISA test 24 h after the treatment of cell co-cultures exposed to LPS. The levels of the cytokine were significantly reduced either by CsA_rm or by formulated CsA compared to the vehicle (Figure 6). Mannitol, used as an excipient in the formulation, also showed a slight anti-inflammatory effect albeit not statistically significant, as already reported in vivo [51]. The results confirm that through the spray-drying process, it was possible to construct highly respirable particles with improved dissolution rates, preserving the CsA anti-inflammatory effect. An inhaled powder of CsA, therefore, represents a favourable therapeutic strategy to avoid the triggering of a vigorous immune reaction in the lungs. Consequently, this action would limit the production of cytokines and their consequent spillover into the circulatory system, preventing the systemic cytokine storm.

### 3.4. In Vitro Anti-Viral Efficacy against SARS-CoV-2

As mentioned before, CsA has been shown to have a direct inhibitory effect on the replication of different types of coronaviruses, including SARS-CoV-2. For this purpose, orally administrated CsA has also been the subject of clinical trials, reporting positive results on the survival of patients affected by COVID-19 [46,52]. Moreover, to date, a further ten clinical trials are ongoing, although the results have not yet become available, indicating the high interest in CsA for the treatment of this disease.

In light of these considerations, the last part of the study explored the inhibition activity of CsA_M20 powder on viral replication in Vero E6 cells in comparison to the CsA_rm. Furthermore, different types of treatment (pre-treatment, post-treatment, or simultaneous regimen) were adopted to assess the more effective one to contain the virus. 

**Figure 7:**
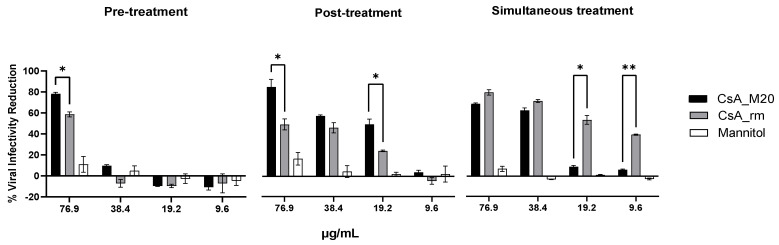
In vitro SARS-CoV-2 Omicron BA.1 infectivity reduction produced by CsA_rm, CsA_M20 or mannitol. Pre-treatment: one hour before infection. Post-treatment: two hours after infection. Simultaneous treatment: cells were infected and treated with the powders at the same time. The mannitol concentrations corresponding to CsA formulation at 76.9, 38.4, 19.2, and 9.6 µg/mL were respectively 15.4, 7.7, 3.8, and 1.9 µg/mL. Data were analysed with two-way analysis of variance (ANOVA) (* *p* < 0.05; ** *p* < 0.01; CsA_M20 vs. CsA_rm).

The infected cells were treated with CsA_rm, CsA_M20, or mannitol powders applied according to the different treatments. Figure 7 illustrates the virus infectivity reduction in relation to the CsA concentrations applied. The effect of mannitol alone was as well assessed since it is a component of the engineered CsA powder. The range of CsA concentrations investigated was selected according to the one proposed by de Wilde et al. [20]. A 100% viral infectivity reduction corresponds to the maximal reduction in the viral load.

During the pre-treatment, only the highest CsA concentration applied (76.9 µg/mL) showed an antiviral effect. The reduction of viral infectivity was 78% when the drug was formulated as a spray-dried powder and was statistically superior to the raw material, which reduced the infection by 58%. At lower concentrations, CsA did not have any relevant antiviral effect. Similarly, mannitol did not produce inhibitory effects at any of the tested concentrations. At the lowest concentrations (19.2 and 9.6 µg/mL) of all of the treatments, even greater viral growth was observed in the treated samples compared to the control; this is identified by the negative value of the infectivity percentage. To interpret the data, it should be mentioned that the cell culture medium containing CsA was replaced with fresh medium before applying the virus; therefore, the drug that interacted with the pathogen replication was only the fraction that was internalised by the cell. The fact that CsA_M20 has superior efficacy to CsA_rm could be due to the higher solubility of these composite particles, possibly increasing the host intracellular concentration of the drug where the virus was replicating. These positive inhibition results show that CsA is active not only against SARS-CoV, as shown in 2011 by de Wilde et al. [20], but also on the SARS type CoV-2 responsible for the current sanitary emergency. It was demonstrated that CsA treatment rendered the virus RNA and protein synthesis almost undetectable [20]. In parallel, the reduction of cyclophilins did not interfere with the SARS-CoV replication. Finally, a further blocking mechanism has been recently in silico demonstrated: through molecular docking, CsA was able to bind and block two membrane proteins (TMPRSS2 and CTSL) necessary for SARS-CoV-2 to penetrate the host cell [53].

Differently from the pre-treatment condition, in the post-treatment regimen, the viral inhibitory activity was present for all CsA concentrations tested except for the lowest one. Furthermore, the CsA_M20 powder was always more effective than the CsA_rm, although statistically superior only at the concentration of 19.2 and 76.9 μg/mL. Mannitol, as in the previous case, showed a slight activity of reducing infectivity. With regard to the adopted protocol, in this case, the treatment was applied after the virus had been allowed to absorb and then removed from the culture. Therefore, as in the case of the pre-treatment, the block of the virus infection presumably took place within the host cell, where the viruses remained after washing resided. The engineered CsA powder had, in these conditions, superior efficacy likely due to its enhanced dissolution, leading to a higher amount of the drug entering the host cell where the virus was replicating.

In the simultaneous treatment, the CsA_M20 and CsA_rm powders performed similarly at the two highest concentrations tested where the inhibition reached 75–80%. This trend changed at 19.2 and 9.6 µg/mL, at which only raw CsA showed an antiviral effect of 30% significantly higher than that of CsA_M20 (5%). This was the only experimental protocol in which the cells were exposed to the virus simultaneously with the treatment, therefore the only situation in which the drug–virus interaction took place both in the extracellular compartment and subsequently intracellularly. The inhibition data of the CsA_rm highlight that an interaction may occur between drug suspension and the virus, which does not happen in the case of the more soluble CsA_M20 powder. In fact, the members of the *Coronaviridae* family possess a phospholipid envelope, therefore an interaction between the pure CsA_rm and the viral membrane would be possible. It is known that CsA binds lipid membranes following the classic hydrophobic effect and that CsA affects the membranes in a concentration-dependent manner by the perturbation of the organisation of fatty chains [54]. Hence, it can be hypothesised that in the case of the CsA_rm, the solid particles create a concentration at the particle–virus interface close to saturation, higher than that generated by the CsA_M20 solubilised in the medium. This difference could explain the high ability to interact with the cell membrane of the virus. Furthermore, the presence of solid particles could represent a further obstacle to infection as they act as a physical barrier and reduce the surface area available for virus adsorption. In contrast, CsA_M20, which was successfully dissolved in the medium, could little hinder the interaction between the virus and the host cell membrane. In this regard, the creation of a polymeric barrier is exploited as a system to inhibit virus–cell interaction by numerous commercially available nasal sprays to antagonise the infection.

In summary, the most effective treatment regimens were post-infection or simultaneous infection treatment. In both cases, the infectivity of SARS-CoV-2 was reduced and in the case of post-treatment, more efficiently by the CsA_M20 powder than the raw material. This post-infection approach is also the most plausible considering that pharmacological treatment commonly follows and does not simultaneously accompany the entry of the virus. Moreover, the raw material, although effective, cannot be administered as such due to its low respirability. At variance, the prophylactic treatment, despite the in vitro data, has not been proven to be effective except at the highest concentration tested, probably because in cases of treatment with a lower dosage, an effective drug concentration is not internalised and retained by the cells.

## 4. Conclusions

The work demonstrated that, through the modulation of mannitol and ethanol, it was possible to achieve an inhalation powder with high respirability (FPF of 66.5%) and improved CsA release (WDT of 59.5 min). This aspect is of crucial importance considering that CsA has a very low oral bioavailability and therefore a rapid lung release would be extremely advantageous to obtain high pulmonary exposure. 

Besides the fact that the inhalation powder developed could represent an advantageous strategy in the prevention of lung transplant rejection, the collected findings provide strong in vitro evidence that this therapeutic approach could be efficient in the reduction of SARS-CoV-2 infectivity, especially as a post-infection treatment. CsA_M20 powder, applied to cells one hour after contact with the virus, was able to inhibit its replication by 93%. Finally, the CsA-engineered powder showed an anti-inflammatory effect in terms of IL-6 reduction that could also be useful in containing the COVID-19 cytokine storm in the lungs.

## Figures and Tables

**Table 1:** Composition of powders studied according to the DOE with three factors at two levels and three centre points (*). M = mannitol; G = glycine. Each experimental point was replicated to calculate the experimental error.

Powder (#, Code)	Factor A: Mannitol (% *w*/*w*)	Factor B: Glycine (% *w*/*w*)	Factor C: Ethanol (% *v*/*v*)
1 (CsA_M15) *	15	2.5	52.5
2 (CsA_M10G)	10	5	45
3 (CsA_M20G)	20	5	60
4 (CsA_M10)	10	0	60
5 (CsA_M20)	20	0	45
6 (CsA_M20G)	20	5	60
7 (CsA_M15) *	15	2.5	52.5
8 (CsA_M15) *	15	2.5	52.5
9 (CsA_M20)	20	0	45
10 (CsA_M10G)	10	5	45
11 (CsA_M10)	10	0	60

**Table 2:** Values of the CQAs investigated for the eleven CsA spray-dried powders: yield of the production process, respirable fraction (RF) < 5 µm, loss on drying (LOD), and time parameter of Weibull equation (time for 63.2% of CsA dissolved from composite powders) indicated as WDT. Data presented as n = 3, mean value ± SD.

Batch	Yield	RF (%)	LOD (%)	WDT (min)
1 (CsA_M15) *	55.7 ± 2.6	64.5 ± 6.3	2.85 ± 0.20	89.7 ± 7.4
2 (CsA_M10G)	55.4 ± 4.1	71.6 ± 1.6	3.41 ± 0.31	99.9 ± 4.8
3 (CsA_M20G)	59.1 ± 7.9	72.0 ± 4.1	1.96 ± 0.12	116.2 ± 8.3
4 (CsA_M10)	61.1 ± 3.8	64.5 ± 1.6	1.75 ± 0.33	109.6 ± 5.7
5 (CsA_M20)	59.5 ± 6.5	70.9 ± 0.4	2.04 ± 0.24	61.7 ± 0.2
6 (CsA_M20G)	56.9 ± 3.2	61.7 ± 0.3	1.98 ± 0.14	138.5 ± 6.7
7 (CsA_M15) *	62.6 ± 6.0	65.4 ± 2.4	2.52 ± 0.55	90.8 ± 14.1
8 (CsA_M15) *	65.0 ± 3.2	70.5 ± 8.7	2.45 ± 0.14	89.7 ± 13.7
9 (CsA_M20)	61.6 ± 4.1	59.9 ± 3.1	2.12 ± 0.31	57.7 ± 0.2
10 (CsA_M10G)	61.1 ± 5.1	61.3 ± 4.4	3.06 ± 0.42	110.6 ± 1.5
11 (CsA_M10)	65.3 ± 2.9	56.6 ± 2.9	1.75 ± 0.25	119.3 ± 10.1

* = central points of the DOE.

**Table 3:** Probability values for the model terms relating to selected CQAs. RF = respirable fraction; LOD = loss on drying; WDT = Weibull dissolution time. The model was significant at *p* < 0.05 and highlighted in bold.

Term	Yield	RF	LOD	WDT
Model	0.425	0.748	**0.033**	**0.006**
R^2^	0.312	0.187	0.944	0.934
Mannitol	0.643	0.580	**0.006**	0.107
Glycine	0.152	0.443	**0.006**	**0.015**
Ethanol	0.568	0.636	**0.0004**	**0.004**

**Table 4:** Aerodynamic characterisation of the CsA_M20 powder at time zero and during the stability investigation in standard and accelerated conditions (n = 3, mean value ± SD).

	Metered Dose (mg)	Emitted Dose (mg)	MMAD (µm)	FPD (mg)	FPF (%)	EFPD (mg)	EFPF (%)
CsA_M20 0 time	14.8 ± 0.1	13.2 ± 0.3	2.97 ± 0.12	8.80 ± 0.18	66.5 ± 2.6	3.61 ± 0.08	27.3 ± 1.2
CsA_M20 1 month 25 °C	14.7 ± 0.3	13.0 ± 0.5	2.58 ± 0.03	9.35 ± 0.46	71.4 ± 0.9	4.24 ± 0.13	32.4 ± 0.2
CsA_M20 1 month 40 °C	15.3 ± 0.3	12.6 ± 0.2	2.38 ± 0.12	8.82 ± 0.41	69.7 ± 4.4	4.38 ± 0.29	34.6 ± 2.8
CsA_M20 3 months 25 °C	14.6 ± 0.3	12.6 ± 0.3	2.61 ± 0.10	9.47 ± 0.22	74.7 ± 0.1	4.11 ± 0.09	32.4 ± 1.5
CsA_M20 3 months 40 °C	15.0 ± 0.3	12.5 ± 0.9	2.58 ± 0.15	8.55 ± 0.85	68.3 ± 2.0	3.75 ± 0.04	30.1 ± 1.8

## Data Availability

The data presented in this study are available on request from the corresponding author.

## Supplementary Materials

The following supporting information can be downloaded at: https://www.mdpi.com/article/10.3390/pharmaceutics15031023/s1, Figure S1. XRPD scan of spray-dried powders and raw materials; Figure S2. XRPD scan of mannitol raw material (light blue), CsA raw material (green), CsA_M20 powder at time 0 (blue) and after 6 months (red); Figure S3. DSC scan of: CsA raw material (CsA_rm) in black; CsA pure spray dried: (CsA_SD) in red; Figure S4. DSC scan of mannitol spray-dried (red) and mannitol raw material (black); Figure S5. CsA_M20 powder at time 0 after production (red) and CsA_M20 after 6 months at room temperature (black). References [33,55,56,57,58,59,60] are cited in the supplementary materials.

## References

[1] Fahr A. (1993). Cyclosporin Clinical Pharmacokinetics. Clin. Pharmacokinet..

[2] Forsythe P., Paterson S. (2014). Ciclosporin 10 Years on: Indications and Efficacy. Vet. Rec..

[3] Tissot A., Danger R., Claustre J., Magnan A., Brouard S. (2019). Early Identification of Chronic Lung Allograft Dysfunction: The Need of Biomarkers. Front. Immunol..

[4] Barr M., Chaparro C., Corris P., Doyle R., Glanville A., Klepetko W., Mcneil K., Orens J., Singer L., Trulock E. (2001). Bronchiolitis Obliterans Syndrome 2001: An Update of the Diagnostic. J. Heart Lung Transplant..

[5] Boehler A., Estenne M. (2003). Post-Transplant Bronchiolitis Obliterans. Eur. Respir. J..

[6] Chan C. (1984). Side Effects of Systemic Cyclosporine in Patients Not Undergoing Transplantation. Am. J. Med..

[7] Parekh K., Trulock E., Patterson G.A. (2004). Use of Cyclosporine in Lung Transplantation. Transplant. Proc..

[8] Kolars J.C., Awni W.M., Merion R.M., Watkins P.B. (1991). First-Pass Metabolism of Cyclosporin by the Gut. Lancet.

[9] Wu C.Y., Benet L.Z., Hebert M.F., Gupta S.K., Rowland M., Gomez D.Y., Wacher V.J. (1995). Differentiation of Absorption and First-Pass Gut and Hepatic Metabolism in Humans: Studies with Cyclosporine. Clin. Pharmacol. Ther..

[10] Bennani Rtel M., Ternant D., Büchler M., El Hassouni M., Khabbal Y., Achour S., Sqalli T. (2020). Food and Lipid Intake Alters the Pharmacokinetics of Cyclosporine in Kidney Transplants. Fundam. Clin. Pharmacol..

[11] Mendonza A.E., Gohh R.Y., Akhlaghi F. (2008). Blood and Plasma Pharmacokinetics of Ciclosporin in Diabetic Kidney Transplant Recipients. Clin. Pharmacokinet..

[12] Taylor W.J., Robinson J.D., Burckart G.J., Canafax D.M., Yee G.C. (2007). Cyclosporine Monitoring. Ann. Pharmacother..

[13] Sato H., Suzuki H., Yakushiji K., Wong J., Seto Y., Prud’homme R.K., Chan H.K., Onoue S. (2016). Biopharmaceutical Evaluation of Novel Cyclosporine A Nano-Matrix Particles for Inhalation. Pharm. Res..

[14] Corcoran T.E., Smaldone G.C., Dauber J.H., Smith D.A., McCurry K.R., Burckart G.J., Zeevi A., Griffith B.P., Iacono A.T. (2004). Preservation of Post-Transplant Lung Function with Aerosol Cyclosporin. Eur. Respir. J..

[15] Groves S., Galazka M., Johnson B., Corcoran T., Verceles A., Britt E., Todd N., Griffith B., Smaldone G.C., Iacono A. (2010). Inhaled Cyclosporine and Pulmonary Function in Lung Transplant Recipients. J. Aerosol Med. Pulm. Drug Deliv..

[16] Iacono A., Wijesinha M., Rajagopal K., Murdock N., Timofte I., Griffith B., Terrin M. (2019). A Randomised Single-Centre Trial of Inhaled Liposomal Cyclosporine for Bronchiolitis Obliterans Syndrome Post-Lung Transplantation. ERJ Open Res..

[17] Nagy P.D., Wang R.Y., Pogany J., Hafren A., Makinen K. (2011). Emerging Picture of Host Chaperone and Cyclophilin Roles in RNA Virus Replication. Virology.

[18] Tanaka Y., Sato Y., Sasaki T. (2013). Suppression of Coronavirus Replication by Cyclophilin Inhibitors. Viruses.

[19] Fenizia C., Galbiati S., Vanetti C., Vago R., Clerici M., Tacchetti C., Daniele T. (2021). Cyclosporine A Inhibits Viral Infection and Release as Well as Cytokine Production in Lung Cells by Three SARS-CoV-2 Variants. Microbiol. Spectr..

[20] de Wilde A.H., Zevenhoven-Dobbe J.C., van der Meer Y., Thiel V., Narayanan K., Makino S., Snijder E.J., van Hemert M.J. (2011). Cyclosporin A Inhibits the Replication of Diverse Coronaviruses. J. Gen. Virol..

[21] Molyvdas A., Matalon S. (2020). Cyclosporine: An Old Weapon in the Fight against Coronaviruses. Eur. Respir. J..

[22] Jayk Bernal A., Gomes da Silva M.M., Musungaie D.B., Kovalchuk E., Gonzalez A., Delos Reyes V., Martín-Quirós A., Caraco Y., Williams-Diaz A., Brown M.L. (2022). Molnupiravir for Oral Treatment of Covid-19 in Nonhospitalized Patients. N. Engl. J. Med..

[23] Shrestha N.K., Burke P.C., Nowacki A.S., Terpeluk P., Gordon S.M. (2022). Molnupiravir and Nirmatrelvir-Ritonavir: Oral COVID Antiviral Drugs Louis. Clin. Infect. Dis..

[24] Lamb Y.N. (2022). Nirmatrelvir Plus Ritonavir: First Approval. Drugs.

[25] Lin H.X.J., Cho S., Meyyur Aravamudan V., Sanda H.Y., Palraj R., Molton J.S., Venkatachalam I. (2021). Remdesivir in Coronavirus Disease 2019 (COVID-19) Treatment: A Review of Evidence. Infection.

[26] Sinha S., Rosin N.L., Arora R., Labit E., Jaffer A., Cao L., Farias R., Nguyen A.P., de Almeida L.G.N., Dufour A. (2022). Dexamethasone Modulates Immature Neutrophils and Interferon Programming in Severe COVID-19. Nat. Med..

[27] Ely E.W., Ramanathan K., Antognini D., Combes A., Paden M., Zakhary B., Ogino M., Maclaren G., Brodie D. (2020). Efficacy and Safety of Baricitinib plus Standard of Care for the Treatment of Critically Ill Hospitalised Adults with COVID-19 on Invasive Mechanical Ventilation or Extracorporeal Membrane Oxygenation: An Exploratory, Randomised, Placebo-Controlled Trial. Lancet Respir. Med..

[28] Gupta A., Gonzalez-Rojas Y., Juarez E., Crespo Casal M., Moya J., Falci D.R., Sarkis E., Solis J., Zheng H., Scott N. (2021). Early Treatment for Covid-19 with SARS-CoV-2 Neutralizing Antibody Sotrovimab. N. Engl. J. Med..

[29] Berton P., Mishra M.K., Choudhary H., Myerson A.S., Rogers R.D. (2019). Solubility Studies of Cyclosporine Using Ionic Liquids. ACS Omega.

[30] Sonvico F., Chierici V., Varacca G., Quarta E., D’Angelo D., Forbes B., Buttini F. (2021). Respicelltm: An Innovative Dissolution Apparatus for Inhaled Products. Pharmaceutics.

[31] Langenbucher F. (1972). Letters to the Editor: Linearization of Dissolution Rate Curves by the Weibull Distribution. J. Pharm. Pharmacol..

[32] Li Y., Su R., Chen J., Li Y., Su R., Chen J. (2020). Co-Culture Systems of Drug-Treated Acute Myeloid Leukemia Cells and T Cells for In Vitro and In Vivo Study. STAR Protoc..

[33] Burnett L., McQueen M.J., Jonsson J.J., Torricelli F. (2007). IFCC Position Paper: Report of the IFCC Taskforce on Ethics: Introduction and Framework. Clin. Chem. Lab. Med..

[34] Sato H., Kawabata Y., Yuminoki K., Hashimoto N., Yamauchi Y. (2012). Comparative Studies on Physicochemical Stability of Cyclosporine A-Loaded Amorphous Solid Dispersions. Int. J. Pharm..

[35] Yamasaki K., Kwok P.C.L., Fukushige K., Prud’Homme R.K., Chan H.K. (2011). Enhanced Dissolution of Inhalable Cyclosporine Nano-Matrix Particles with Mannitol as Matrix Former. Int. J. Pharm..

[36] Leung S.S.Y., Wong J., Guerra H.V., Samnick K., Prud’homme R.K., Chan H.K. (2017). Porous Mannitol Carrier for Pulmonary Delivery of Cyclosporine A Nanoparticles. AAPS J..

[37] Iacono A.T., Johnson B.A., Grgurich W.F., Youssef J.G., Corcoran T.E., Seiler D.A., Dauber J.H., Smaldone G.C., Zeevi A., Yousem S.A. (2006). A Randomized Trial of Inhaled Cyclosporine in Lung-Transplant Recipients. N. Engl. J. Med..

[38] Iacono A. (2021). Capitalizing on the Concept of Local Immune Suppression by Inhalation for Lung Transplant Recipients. Am. J. Transplant..

[39] Neurohr C., Kneidinger N., Ghiani A., Monforte V., Knoop C., Jaksch P., Parmar J., Ussetti P., Sole A., Quernheim J.M.- (2022). A Randomized Controlled Trial of Liposomal Cyclosporine A for Inhalation in the Prevention of Bronchiolitis Obliterans Syndrome Following Lung Transplantation. Am. J. Transplant..

[40] Behr J., Zimmermann G., Baumgartner R., Leuchte H., Neurohr C., Brand P., Herpich C., Sommerer K., Seitz J., Menges G. (2009). Lung Deposition of a Liposomal Cyclosporine a Inhalation Solution in Patients after Lung Transplantation. J. Aerosol Med. Pulm. Drug Deliv..

[41] Wu X., Zhang W., Hayes D., Mansour H.M. (2013). Physicochemical Characterization and Aerosol Dispersion Performance of Organic Solution Advanced Spray-Dried Cyclosporine A Multifunctional Particles for Dry Powder Inhalation Aerosol Delivery. Int. J. Nanomed..

[42] Suzuki H., Ueno K., Mizumoto T., Seto Y., Sato H., Onoue S. (2017). Self-Micellizing Solid Dispersion of Cyclosporine A for Pulmonary Delivery: Physicochemical, Pharmacokinetic and Safety Assessments. Eur. J. Pharm. Sci..

[43] Yang T.T., Wen B.F., Liu K., Qin M., Gao Y.Y., Ding D.J., Li W.T., Zhang Y.X., Zhang W.F. (2018). Cyclosporine A/Porous Quaternized Chitosan Microspheres as a Novel Pulmonary Drug Delivery System. Artif. Cells Nanomed. Biotechnol..

[44] Anderson S., Atkins P., Bäckman P., Cipolla D., Clark A., Daviskas E., Disse B., Entcheva-Dimitrov P., Fuller R., Gonda I. (2022). Inhaled Medicines: Past, Present, and Future. Pharmacol. Rev..

[45] Belotti S., Rossi A., Colombo P., Ruggero B., Rekkas D., Politis S., Colombo G., Balducci A.G., Buttini F. (2015). Spray-dried amikacin sulphate powder for inhalation in cystic fibrosis patients: The role of ethanol in particle formation. Eur. J. Pharm. Biopharm..

[46] Guisado-Vascoa P., Valderas-Ortegab S., Carralòn-Gonzalez M.M., Roda-Santacruzc A., Lucia Gonzalez-CortijoGabriel G.S.-F., Roda-santacruz A., Gonz L., Martí-ballesteros E.M., Luque-pinilla J.M., Almagro-casado E. (2020). Clinical Characteristics and Outcomes among Hospitalized Adults with Severe COVID-19 Admitted to a Tertiary Medical Center and Receiving Antiviral, Antimalarials, Glucocorticoids, or Immunomodulation with Tocilizumab or Cyclosporine: A Retrospective O. EClinicalMedicine.

[47] Buttini F., Quarta E., Allegrini C., Lavorini F. (2021). Understanding the Importance of Capsules in Dry Powder Inhalers. Pharmaceutics.

[48] Wheeler D.S., Misumi K., Walker N.M., Vittal R., Combs M.P., Aoki Y., Braeuer R.R., Lama V.N. (2021). Interleukin 6 Trans-Signaling Is a Critical Driver of Lung Allograft Fibrosis. Am. J. Transplant..

[49] Rose-John S., Winthrop K., Calabrese L. (2017). The Role of IL-6 in Host Defence against Infections: Immunobiology and Clinical Implications. Nat. Rev. Rheumatol..

[50] Zhou J., He W., Liang J., Wang L., Yu X., Bao M., Liu H. (2021). Association of Interleukin-6 Levels with Morbidity and Mortality in Patients with Coronavirus Disease 2019 (COVID-19). Jpn. J. Infect. Dis..

[51] Reisi Nassab P., Blazsó G., Nyári T., Falkay G., Szabó-Révész P. (2008). In Vitro and In Vivo Investigations on the Binary Meloxicam-Mannitol System. Pharmazie.

[52] Blumberg E.A., Noll J.H., Tebas P., Fraietta J.A., Frank I., Marshall A., Chew A., Veloso E.A., Carulli A., Rogal W. (2022). A Phase I Trial of Cyclosporine for Hospitalized Patients with COVID-19. JCI Insight.

[53] Prasad K., Ahamad S., Kanipakam H., Gupta D., Kumar V. (2021). Simultaneous Inhibition of SARS-CoV-2 Entry Pathways by Cyclosporine. ACS Chem. Neurosci..

[54] Czogalla A. (2009). Oral Cyclosporine A—The Current Picture of Its Liposomal and Other Delivery Systems. Cell. Mol. Biol. Lett..

[55] Ammerman N., Beier-Sexton M., Azad A. (2009). Growth and Maintenance of Vero Cell Lines. Curr. Protoc. Microbiol..

[56] Lei C., Yang J., Hu J., Sun X. (2021). On the Calculation of TCID50 for Quantitation of Virus Infectivity. Virol. Sin..

[57] Reed L.J., Muench H. (1938). A Simple Method of Estimating Fifty per Cent Endpoints. Am. J. Epidemiol..

[58] Jiang X., Zhao Y., Guan Q., Xiao S., Dong W., Lian S., Zhang H., Liu M., Wang Z., Han J. (2022). Amorphous Solid Dispersions of Cyclosporine A with Improved Bioavailability Prepared via Hot Melt Extrusion: Formulation, Physicochemical Characterization, and in Vivo Evaluation. Eur. J. Pharm. Sci..

[59] Benetti A.A., Bianchera A., Buttini F., Bertocchi L., Bettini R. (2021). Mannitol Polymorphs as Carrier in Dpis Formulations: Isolation Characterization and Performance. Pharmaceutics.

[60] Adi H., Young P.M., Chan H.K., Agus H., Traini D. (2010). Co-Spray-Dried Mannitol-Ciprofloxacin Dry Powder Inhaler Formulation for Cystic Fibrosis and Chronic Obstructive Pulmonary Disease. Eur. J. Pharm. Sci..

