# Supplementary material for: An Enhanced Dissolving Cyclosporin-A Inhalable Powder Efficiently Reduces SARS-CoV-2 Infection In Vitro
Source: Pharmaceutics. 2023 Mar 22;15(3):1023. doi: 10.3390/pharmaceutics15031023 (PMC10055879; doi:10.3390/pharmaceutics15031023)
Supplement: Supplementary file 1 [file pharmaceutics-15-01023-s001.zip › pharmaceutics-2239569-supplementary.pdf]

Supplementary Materials

1. Details of the *in vitro* analysis on viral infectivity reduction on Vero E6

1.1 *In vitro cell culture set-up*

Vero E6 cell cultures (American Type Culture Collection [ATCC] CRL-1586) were grown in Minimum Essential Medium (MEM) supplemented with 2 mM L-glutamine, 100 U/mL penicillin, 100 μg/mL streptomycin (complete culture medium) and 10% heat-inactivated fetal bovine serum (FBS), as recommended [55]. Cells were incubated at 37°C in a humidified, 5% CO_2 atmosphere-enriched chamber until use. For compound treatment studies, cells were seeded in 96-well plates and cultured in MEM containing 2% FBS. Cell culture medium and supplements were purchased from EuroClone (Milan, Italy).

1.2 *Virus propagation and titration*

The viral strain was isolated from a residual clinical specimen conferred to the Unit of Microbiology, Greater Romagna Area Hub Laboratory, Cesena, Italy, for routine diagnostic purposes and sequenced as part of the project for monitoring the prevalence and distribution of SARS-CoV-2 variants in Italy, promoted by the Italian Institute of Public Health (ISS). Before being used for this study, the sample underwent an anonymization procedure, to adhere to the regulations issued by the local Ethical Board (AVR-PPC P09, rev.2; based on Burnett et al., 2007 [33]). In brief, 500 μL of clinical specimens was used to infect the Vero E6 cell monolayer at confluency. After one-hour adsorption, the culture was maintained in 2% FBS MEM and incubated for 72 hours. The viral strain was titrated using the endpoint dilution method [56]. In brief, serial 10-fold dilutions (from 10-1 to 10-10) in 2% FBS MEM were used to infect confluent monolayers of cells in a 96-well plate. After 72 hours cells were fixed and stained using a 4% formaldehyde solution in crystal violet. The absence or presence of cytopathic effect at each dilution was assessed by comparison of each well with virus control and cell control wells. Viral titres, expressed as TCID (Median Tissue Culture Infectious Dose) 50/mL, were calculated with the Reed and Muench formula based on eight replicates for dilution [57].

1.3 *Preparation of the sample for the PCR analysis and details of amplification*

For the PCR quantification, 15 μL of the sample was diluted 1:4 in 45 μL of RNase-free water in a 96-well PCR plate and hence 5 μL of the dilution was transferred to another plate with 16 μL of PCR master mix, containing 5 μL of MOM (MuDT Oligo Mixture, with dNTPs, oligos, primers and TaqMan 5′ fluorophore/3′ Black Hole Quencher probes), 5 μL of enzymes, 5 μL of RNase-free water and 1 μL of exogenous internal control for every reaction.

The assay was run on a CFX96 real-time thermal cycler (Bio-Rad, Feldkirchen, Germany). The

amplification process includes cDNA denaturation at 95 °C for 10 seconds, primers annealing at 60 °C for 15 seconds and elongation at 72 °C for 10 seconds (44 cycles). Results analysis and targets quantification were performed with 2019-nCoV Viewer from Seegene Inc.

2. Solid State Analysis of CsA spray-dried powders

2.1 X-Ray Powder Diffraction

This analysis was assessed using a MiniFlex diffractometer (Rigaku, Tokyo, Japan) with a 30 kV Cu Kα radiation (λ = 1.5418 Å). The powder was placed in the aluminium sample holder and subsequently flattened to obtain a smooth surface. The goniometer was set at a scanning rate of 1.5° min^{-1} (step size = 0.05°) over the 2 θ range 5–35°.

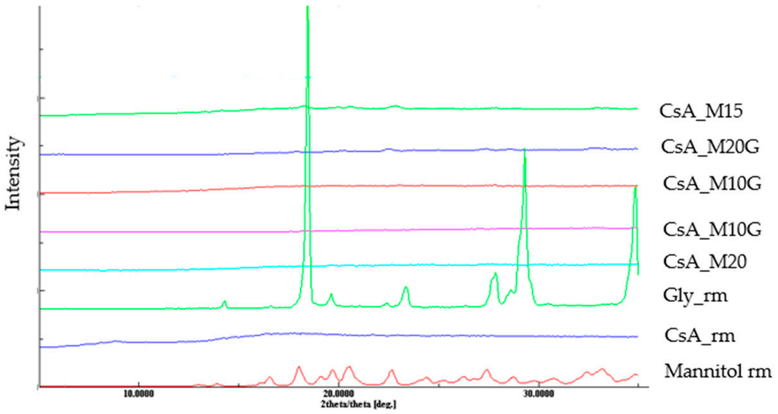

Figure S1. XRPD scan of spray-dried powders and raw materials.

The influence of different concentrations of ethanol and glycine on the solid state of the spray-dried powders was evaluated by XRPD (Figure 1S). After the spray drying process, the powders do not present the characteristic peaks of raw mannitol or glycine regardless of the ratio of the ingredients used. For this reason, the difference in solubility among powders is not attributable to the solid state of the particles.

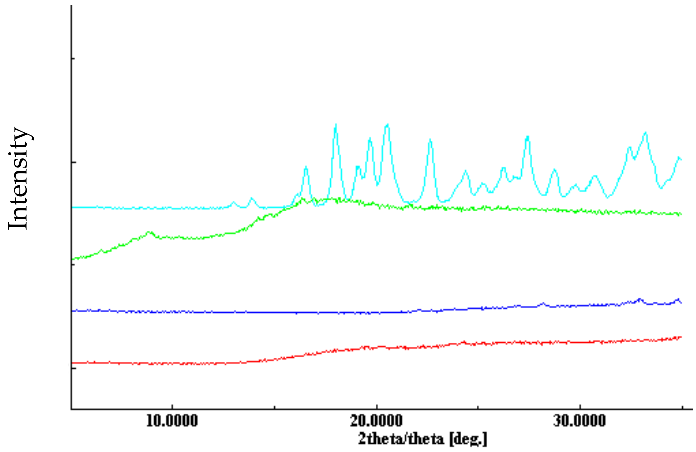

Figure S2. XRPD scan of mannitol raw material (light blue), CsA raw material (green), CsA_M20 powder at time 0 (blue) and after 6 months (red).

The XRPD patterns in Figure 2S indicate that CsA_rm was amorphous, while mannitol raw material was crystalline. Moreover, the CsA_M20 powders were amorphous both at time 0 and 6 months after production, and no characteristic peaks of the crystalline state were observed.

2.2 *Differential Scanning Calorimetry Analysis*

DSC scan was performed to characterize the spray-dried powders and the single excipients before the drying process. It is known that it is extremely important to assess that the solid state of the material does not change over time to prevent changes in the aerodynamic profile and other fundamental characteristics of the powder. Initially, a DSC analysis of CsA_rm and pure CsA_SD was performed (Figure. 3S). Raw cyclosporine, before and after spray drying, appears to have a similar DSC profile characterized by an initial broad endotherm, between 35 and 100 °C, that was ascribed to the evaporation of the moisture in the samples. This endothermic event was followed by a further endothermic event at 130°C attributable to glassy-rubbery transition as previously reported by Jiang et al., [58].

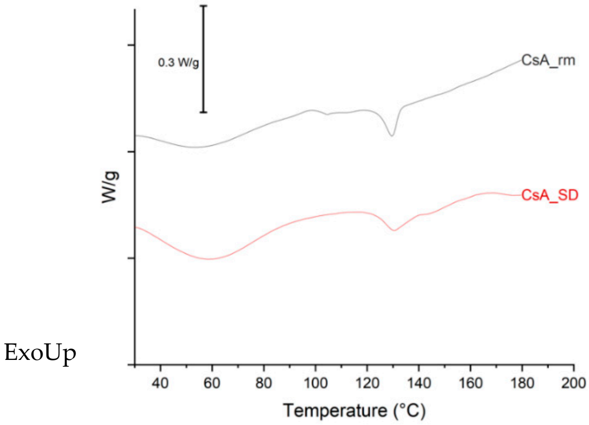

Figure S3. DSC scan of: CsA raw material (CsA_rm) in black; CsA pure spray dried: (CsA_SD) in red.

Subsequently, the DSC traces of mannitol raw material and pure mannitol spray-dried, prepared using the same conditions of solution composition and process parameters of CsA_M20, were compared (Figure 4S).

Both raw mannitol and spray-dried mannitol showed a single endothermic event at 166.78°C and 167.32°C for spray-dried and raw mannitol respectively, in strong agreement with what is reported in the literature in analyses concerning the beta form of mannitol, moreover as previously reported in the literature, spray dried mannitol alone recrystallize in the beta form [59,60].

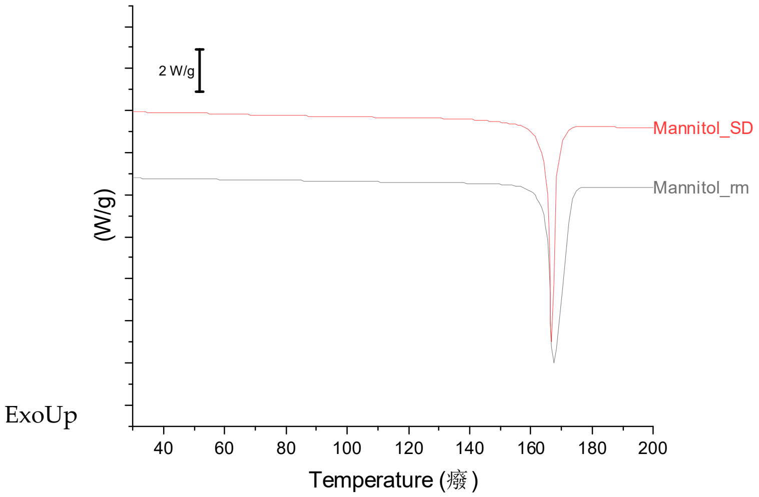

Figure S4. DSC scan of mannitol spray-dried (red) and mannitol raw material (black).

Finally, Figure 5S shows the analysis of the CsA_M20 powder at time zero and after 6 months of storage at room temperature. This powder contains 20% mannitol; it is known that this material can exist in various polymorphic forms (α, β, δ) and that as a pure amorphous material, it tends quickly to convert into the most stable β crystalline form.

The DSC analysis of CsA_M20 showed a similar profile at time zero and after storage of 6 months at room temperature. At time zero, the thermogram showed an initial endothermic peak at 60°C likely due to the glass transition of the material. At 143°C an exothermic peak was observed, interpreted as the recrystallization of the amorphous mannitol followed by an endo-exo event starting at 156°C attributable to the melting of mannitol delta form immediately followed by the recrystallization of the beta form that eventually melted at 168°C [55,56].

As regard the behaviour of the CsA_M20 after 6 months of storage, two slight differences were observed: the initial endothermic peak was anticipated at 45°C and the mannitol exothermic recrystallization event (at 142°C) was more evident. Despite these thermal behaviour modifications, the aerodynamic behaviour and chemical stability of the peptide was not different from time zero,

indicating that this event does not affect the critical characteristics of the product.

This analysis shows how the spray drying process of mannitol with cyclosporine leads to the formation of an amorphous solid, in which mannitol under heating recrystallised in the delta polymorph different to the one of mannitol alone (the beta-crystalline phase).

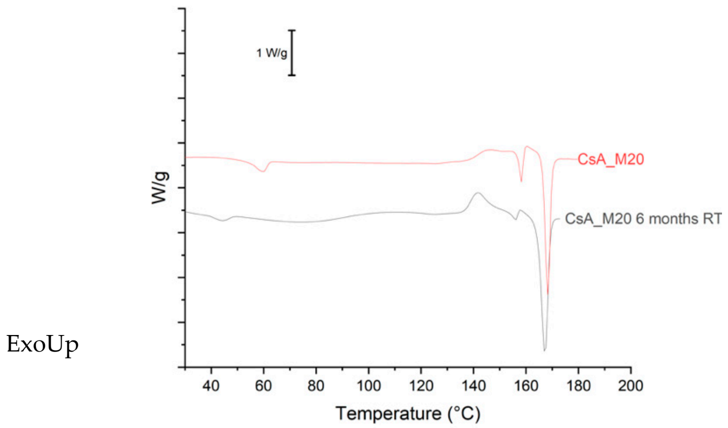

Figure S5. CsA_M20 powder at time 0 after production (red) and CsA_M20 after 6 months at room temperature (black).
